# School-based drug prevention in Saudi Arabia: are physical education teachers prepared to act?

**DOI:** 10.3389/fpubh.2026.1911155

**Published:** 2026-08-19

**Authors:** Abdulrahman I. Alaqil

**Affiliations:** College of Education, Department of Physical Education, King Faisal University, Al-Ahsa, Saudi Arabia

**Keywords:** adolescent substance use, attitudes, beliefs, drug prevention education, knowledge, physical education teachers, school health, teacher preparedness

## Abstract

**Background:**

Around 70% of Saudi Arabia’s drug addicts are under 20, yet drug prevention education (DPE) in Saudi schools remains limited. To address this public health crisis, this study explored whether physical education (PE) teachers can help through school-based prevention initiatives. Data were collected in 2018, providing a baseline assessment at a time when the Saudi school PE workforce was exclusively male.

**Methods:**

A cross-sectional survey was conducted among male PE teachers in Riyadh (*N* = 176) using a validated Arabic questionnaire measuring knowledge, attitudes, beliefs, and self-reported preventive practices. Statistical analyses included descriptive measures, independent-samples *t*-tests, and one-way ANOVAs.

**Results:**

Saudi male PE teachers demonstrated generally adequate knowledge about drug abuse prevention (mean = 5.47/7), though misconceptions persist regarding “gateway” substances such as e-cigarettes and amphetamines. PE teachers expressed strong support for integrating drug prevention into the PE curriculum and believed they could positively influence students’ behavior. Knowledge scores significantly predicted preventive practices, while attitudes and beliefs showed moderate associations with implementation. Despite positive perceptions, most teachers reported limited formal training in DPE and poor awareness of national prevention standards. Many relied on personal judgment rather than evidence-based approaches when discussing substance misuse with students.

**Conclusion:**

Saudi male PE teachers represent a potentially valuable but underutilized resource in DPE. Their close and consistent interaction with students means PE teachers are ideally positioned to promote healthy behaviors, reinforce resilience, and identify early warning signs of substance misuse. However, gaps in DPE training, policy awareness, and evidence-based practice limit their effectiveness. Expanding professional development and incorporating structured prevention education into PE curricula could strengthen school-based public health interventions and contribute to reducing adolescent substance abuse.

## 1 Introduction

Drug abuse is one of the most pressing public health challenges of the twenty-first century. Its consequences extend far beyond the individual, bringing catastrophe to families, communities, and entire health systems (1). Between 2022 and 2023, some 316 million people worldwide admitted to using illegal drugs, marking an increase over the past decade that outpaces population growth (2). Among the most vulnerable populations to the effects of drugs are adolescents, for whom early initiation into substance misuse is associated with significantly higher risks of dependence, mental health disorders, poor academic outcomes, and long-term social dysfunction (2). Preventing adolescent drug use before it begins through DPE, particularly during the school years, is therefore a public health priority of the highest order.

Saudi Arabia is not immune to the global epidemic of drug misuse. Despite strict Islamic laws prohibiting the use of alcohol and illicit substances, substance misuse has continued to escalate, presenting a growing public health challenge with distinctive regional characteristics (3–5). Saudi Arabia’s strict Islamic laws and regulations against drug trafficking and misuse appear to be ineffective against substance use disorder (SUD) trends, including early substance initiation, increased amphetamine use, and rising polysubstance abuse (5). In recent years, Saudi Arabia has emerged as a significant hub for drug trafficking, with illicitly produced amphetamines (commonly known as Captagon) at the forefront, giving rise to widespread use and trade across the Middle East (3, 5). Of particular concern is the disproportionate impact of SUD on the younger population: a scoping review reports that 70% of people who use drugs (PWUD) in Saudi Arabia are aged between 12 and 22 years old (6, 7), underscoring the urgent need for targeted, school-based DPE efforts.

Schools are widely recognized as among the most effective settings for delivering DPE, offering systematic access to large populations of young people during the developmental period when prevention is most impactful (8–10). Within the school environment. Physical Education (PE) teachers occupy a strategically important role in DPE. Unlike subject-specific teachers, PE teachers interact with students across grade levels in settings inherently tied to physical and emotional wellbeing, positioning them as natural health education advocates (11, 12). International literature has consistently identified PE teachers as key agents in effective DPE, capable of positively influencing students’ health knowledge, attitudes, and behaviors (13–15).

The effectiveness of PE teachers in delivering DPE, however, is fundamentally contingent on their own knowledge, attitudes, and beliefs regarding the subject. In the Saudi Arabian context, comparable research highlights that health knowledge among teachers remains frequently inadequate. For example, AlYahya et al. (16) work revealed that male school teachers in Riyadh scored an average of only 41.44% on a first-aid knowledge assessment, with fewer than 15% achieving a “good knowledge” score. Teachers who lack accurate knowledge of a health topic such as DPE are less likely to address it confidently in the classroom, more likely to perpetuate misconceptions, and less equipped to identify and appropriately respond to at-risk students (17, 18).

Despite the severity of Saudi Arabia’s adolescent drug misuse problem and the well-established role of teachers in promoting healthy behaviors among students and delivering DPE, to date, no studies have investigated DPE knowledge and the associated attitudes and beliefs of Saudi Arabian PE teachers. This represents a critical evidence gap: Without a baseline understanding of what PE teachers know, how they feel, and what they believe about DPE, it is not possible to design targeted and effective DPE interventions, whether at the level of pre-service teacher education, in-service continuous professional development (CPD), or school health curriculum policy.

Furthermore, Saudi Arabia’s unique socio-cultural characteristics warrant a dedicated investigation of PE teachers’ role in delivering DPE. Specifically, the cultural and religious stigma surrounding drug use in Saudi Arabia means that drug-related issues remain frequently underreported and under-researched (19). Besides, the absence of a formal health education curriculum in Saudi public schools and the lack of DPE courses offered in pre-service PE teacher preparation programs have created systemic barriers to delivering effective DPE to Saudi Arabian school children. Current efforts at delivering DPE in Saudi schools are likely to be inconsistent, informal, and highly dependent on the motivation and self-directed learning of individual teachers. To address this issue, this study aimed to assess DPE-related knowledge, attitudes, and beliefs among in-service male high school physical education teachers in Riyadh, Saudi Arabia (2018). It is hoped the findings will provide crucial empirical baseline data to facilitate the development of evidence-based, effective DPE curriculum development, teacher training reform, and public health policy changes in the Saudi educational system. Specifically, the study was guided by the following research questions: (1) What is the level of DPE knowledge among in-service male high school PE teachers in Riyadh City? (2) What attitudes do in-service male high school PE teachers hold toward DPE? (3) What beliefs do in-service male high school PE teachers hold regarding DPE, drug prevalence, and referral behavior?

## 2 Method

### 2.1 Study design

A cross-sectional, questionnaire-based research approach was used to assess the DPE-related knowledge, attitudes, and beliefs among in-service male high school PE teachers in Riyadh, Saudi Arabia. This design was selected since it enables the systematic description of a defined professional population’s characteristics at a fixed point in time and has been widely adopted in comparable teacher health-knowledge surveys (16, 18). The study was approved by the Institutional Review Board (IRB) at the University of Northern Iowa (IRB 19-0022); institutional permission was granted by Riyadh Educational District, Ministry of Education, prior to the recruitment of participants. Informed consent was obtained from all participants, and all responses were collected anonymously.

### 2.2 Setting and participants

**Figure 1:**
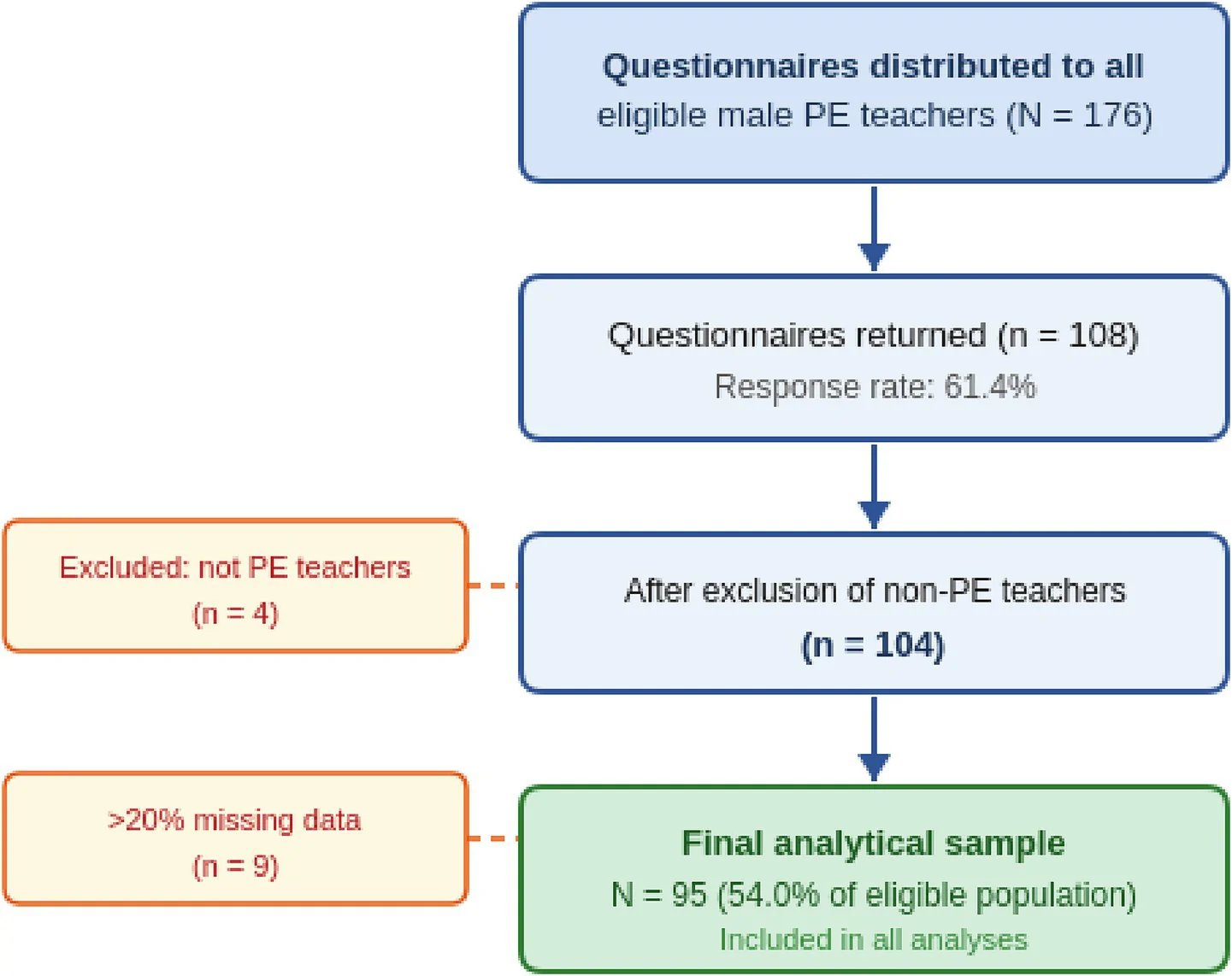
Participant flow diagram showing recruitment and exclusion process.

The Riyadh Educational District administers a total of 208 public and private high schools across five geographical sub-districts of the city: North, South, East, West, and Central. The target population was all in-service male high school PE teachers employed within these five districts at the time of data collection (September to October 2018). The eligibility criteria were (i) male, and (ii) currently employed as a PE teacher at a Riyadh high school. To recruit the sample, a census approach was adopted, and questionnaires were distributed to all 176 eligible participants, ensuring full population coverage. Of the 176 questionnaires distributed, 108 were returned (response rate: 61.4%). Following exclusion of four questionnaires from respondents not currently employed as PE teachers and nine with more than 20% missing data, the final analytical sample comprised 95 participants (see Figure 1). The study was limited to male participants only, since, at the time of data collection (2018), female PE teachers were not yet employed in Saudi public high schools due to the Ministry of Education’s phased implementation of physical education beginning in 2017; sex was therefore not included as an analytical variable.

### 2.3 Instrument

The research instrument was adapted from the validated Drug-Related Knowledge, Attitudes and Beliefs (KAB) survey (20), supplemented with knowledge-related items developed in accordance with the National Health Education Standards (21) and the drug prevention literature (9, 22). The final version of the questionnaire comprised two parts: Part I assessed demographic characteristics and factual DPE knowledge through 20 items (10 true/false/do not know statements on drug pharmacology and Saudi narcotics law, 8 yes/no curriculum goal items, and 2 open-response questions); Part II assessed DPE-related attitudes and beliefs through 8 seven-point Likert scale items (1 = strongly disagree; 7 = strongly agree), 3 DPE-related referral behavior items, and 2 open-response DPE-related questions. Face validity was established through expert panel review. The instrument was translated into Arabic and piloted with eight in-service PE teachers working outside Riyadh prior to the main data collection.

### 2.4 Statistical analysis

SPSS (version 24.0; IBM Corp., Armonk, NY, United States) was used to analyze the data. Descriptive statistics (frequencies, percentages, means, and standard deviations) were computed for all demographic variables and DPE-related outcome scores. Differences in DPE knowledge scores by binary grouping variables were examined using independent-samples t-tests. One-way analysis of variance (ANOVA) was used to compare DPE knowledge scores across variables with three or more categories: years of teaching experience, highest degree attained, school location, and school type, with post-hoc comparisons conducted using the Tukey HSD test where omnibus significance was achieved. Associations between years of experience and DPE knowledge scores were examined using Pearson’s r. All tests were two-tailed, with a significance threshold of *α* = 0.05.

## 3 Result

Of the 176 questionnaires distributed to all in-service male high school PE teachers in Riyadh, 108 were returned (response rate: 61.4%). After excluding four non-PE teachers and nine questionnaires with more than 20% missing data, the final analytical sample comprised 95 participants (54.0% of the total population). The majority held undergraduate degrees (75.8%) and were employed in public schools (78.9%). Years of teaching experience ranged from 0 to > 29 years, with the largest proportion reporting 0–9 years (36.8%), followed by 10–19 years (34.7%) (see Table 1).

**Table 1:** PE teachers’ responses to drug prevention education knowledge items (*N* = 95).

Knowledge item	Correct *n* (%)	Incorrect *n* (%)	Do not know *n* (%)
Addiction only develops when drugs are injected	71 (74.7)	14 (14.7)	10 (10.5)
Tolerance can develop to all drugs, including hashish and amphetamines	68 (71.6)	11 (11.6)	16 (16.8)
There are withdrawal symptoms from alcohol	60 (63.2)	18 (18.9)	17 (17.9)
Amphetamine use can exacerbate mental illness	58 (61.1)	12 (12.6)	25 (26.3)
Amphetamines stimulate the central nervous system	57 (60.0)	14 (14.7)	24 (25.3)
Drug use in Saudi Arabia is a crime	91 (95.8)	2 (2.1)	2 (2.1)
Street drugs are generally pure	55 (57.9)	22 (23.2)	18 (18.9)
Cannabis can be detected in urine for up to 28 days	38 (40.0)	21 (22.1)	36 (37.9)
Amphetamines can be detected in urine for up to 12 days	33 (34.7)	19 (20.0)	43 (45.3)
Possession of cannabis for personal use leads to no more than 2 years in Saudi Arabia	27 (28.4)	25 (26.3)	43 (45.3)

Items are ordered from highest to lowest correct response rate. Composite score computed from all 10 items (1 = correct; 2 = incorrect/do not know).

### 3.1 DPE knowledge

Inferential analyses revealed that teachers who had previously read or learned about DPE achieved significantly higher scores than those who had not (M = 1.39, SD = 0.16 vs. M = 1.53, SD = 0.19; t (88) = −3.45, *p* = 0.001). Neither highest degree attained, school type, nor personal experience with drug-using students significantly predicted DPE knowledge (all *p* > 0.05). Years of teaching experience was a significant predictor of DPE knowledge: those with 0–9 years of teaching experience demonstrated significantly better knowledge of DPE than those with more than 29 years (*F*(3, 86) = 3.3, *p* = 0.023). School location also significantly influenced DPE knowledge, with teachers in the North sub-district outperforming those in the Central sub-district (*F*(4, 85) = 5.1, *p* = 0.001). Group comparisons are summarized in Table 2.

**Table 2:** DPE knowledge scores by demographic and professional characteristics (*N* = 95).

Variable/group	*n*	M (SD)	Statistic	*p*	Sig.
Prior DPE learning			*t*(88) = −3.45	0.001	**
Yes	66	1.39 (0.16)			
No	29	1.53 (0.19)			
Years of teaching experience			*F*(3, 86) = 3.3	0.023	*
0–9 years^†^	35	1.37 (0.15)			
10–19 years	33	1.44 (0.18)			
20–29 years	22	1.46 (0.19)			
>29 years	5	1.63 (0.21)			
School location			*F*(4, 85) = 5.1	0.001	**
North^†^	17	1.29 (0.13)			
East	19	1.41 (0.17)			
South	17	1.43 (0.16)			
West	12	1.44 (0.20)			
Central	30	1.57 (0.19)			
Highest degree attained			*F*(3, 87) = 0.9	0.44	ns
Type of school			*t*(88) = 0.6	0.55	ns
Experience with drug-using students			*t*(88) = 0.4	0.69	ns

M and SD for composite knowledge score (range 1–2; lower = higher knowledge). ^†^Denotes the group with significantly higher knowledge in post-hoc comparisons (Tukey HSD). *p < 0.05. **p < 0.01. ns, not significant.

### 3.2 DPE attitudes

The composite attitude score, derived from the eight Likert scale items (1 = strongly disagree; 7 = strongly agree, with negatively worded items reverse-scored), indicated a broadly neutral attitudinal orientation toward DPE (M = 4.67, SD = 0.95). Teachers who had received prior DPE education held significantly more positive attitudes toward DPE than those who had not (M = 4.84, SD = 0.90 vs. M = 4.29, SD = 0.94; *t*(87) = 2.6, *p* = 0.011). As with knowledge, years of experience (*F*(3, 85) = 3.4, *p* = 0.021) and school location (*F*(4, 84) = 5.4, *p* = 0.001) were the only demographic variables significantly associated with positive attitudes toward DPE, while degree, school type, and experience with drug-using students were not (all *p* > 0.05). Table 3 presents the mean scores and group comparisons for all attitudinal variables.

**Table 3:** DPE attitude scores by demographic and professional characteristics (N = 95).

Variable/group	*n*	M (SD)	Statistic	*p*	Sig.
Overall composite attitude score	95	4.67 (0.95)			
Prior DPE learning			*t*(87) = 2.6	0.011	*
Yes	66	4.84 (0.90)			
No	29	4.29 (0.94)			
Years of teaching experience			*F*(3, 85) = 3.4	0.021	*
0–9 years	35	4.49 (0.92)			
10–19 years	33	4.55 (0.97)			
20–29 years	22	4.91 (0.88)			
>29 years^†^	5	5.31 (0.94)			
School location			*F*(4, 84) = 5.4	0.001	**
North^†^	17	5.12 (0.84)			
East	19	4.73 (0.93)			
West	12	4.68 (0.91)			
South	17	4.43 (0.98)			
Central	30	4.38 (0.97)			
Highest degree attained			*F*(3, 87) = 1.1	0.35	ns
Type of school			*t*(87) = 0.8	0.43	ns
Experience with drug-using students			*t*(87) = 1.2	0.23	ns

Attitude composite score range: 1–7; higher scores = more positive attitude toward DPE. Negatively worded items were reverse-scored. ^†^Denotes group with significantly more positive attitude in post-hoc comparisons (Tukey HSD). *p < 0.05. **p < 0.01. ns, not significant.

### 3.3 DFE beliefs and referral behavior

Teachers’ DPE-related beliefs were assessed across three domains: (i) perceptions of drug prevalence and community context; (ii) curriculum priorities; (iii) professional referral behavior. Regarding perceived drug prevalence, teachers most commonly identified tobacco as the most prevalent drug used by students (44.2%; *n* = 42), followed by hashish (22.1%; *n* = 21) and Captagon (16.8%; *n* = 16). With respect to community context, 54.0% of teachers (*n* = 48) believed that Saudi school communities are tolerant toward drug users, while 53.0% (*n* = 47) agreed that drugs constitute a major issue in their school districts. Regarding curriculum priorities, teachers highly endorsed all eight United States. National Health Education Standards (72.6–94.7%). Table 4 presents the full belief profile across all three domains.

**Table 4:** PE teachers’ beliefs about DPE: community context, drug prevalence, and curriculum priorities (*N* = 95).

Belief Item/category	Agree/yes *n* (%)	Disagree/no *n* (%)	Unsure/no response *n* (%)
A. community context			
Saudi school communities are tolerant toward drug users	48 (54.0)	34 (37.0)	8 (9.0)
Drugs are a major issue in school districts	47 (53.0)	29 (33.0)	11 (13.0)
Government spending on drug prevention is money well spent	76 (80.0)	11 (11.6)	8 (8.4)
DPE should start at the primary school level	79 (83.2)	9 (9.5)	7 (7.4)
All illegal drugs are harmful to health	80 (84.2)	8 (8.4)	7 (7.4)
Drug users are victims rather than criminals	44 (46.3)	37 (38.9)	14 (14.7)
Would avoid teaching a student who uses drugs	29 (30.5)	57 (60.0)	9 (9.5)
Would be bothered to teach a student who uses drugs	32 (33.7)	54 (56.8)	9 (9.5)
B. perceived drug prevalence among students	*n*	%	
Tobacco	42	44.2	
Hashish	21	22.1	
Captagon	16	16.8	
Chewing tobacco	5	5.3	
Alcohol	2	2.1	
C. endorsed curriculum standards (NHES)	*n*	%	
Standard 2: Analyse social/cultural influences on drug behavior	90	94.7	
Standard 1: Comprehend drug prevention concepts	89	93.7	
Standard 5: Use decision-making skills for DPE	87	91.6	
Standard 8: Advocate for community health	85	89.5	
Standards 4 & 7: Communication skills; health-enhancing behaviors	84	88.4	
Standard 6: Goal-setting skills for DPE	79	83.2	
Standard 3: Access valid health information	69	72.6	

Section A: Agree/Yes = combined “agree slightly,” “agree moderately,” and “agree strongly” on the 7-point scale. Section B: Perceived most prevalent drug based on teachers’ classroom experience. Section C: Standards ranked by endorsement rate. NHES, United States. National Health Education Standards; DPE, drug prevention education.

When asked to identify the most appropriate person to whom a student suspected of drug use should be referred, the vast majority of teachers (81.1%; *n* = 77) cited a school counselor, with only 10.5% (*n* = 10) indicating they would attempt to manage the situation themselves. Among the 60 teachers (63.2%) who had personally encountered a drug-using student, the most common response was providing individual counseling directly (51.0%; *n* = 28). Interestingly, 22.0% (*n* = 12) reported taking no formal action. Only one teacher (2.0%) had referred a student to a specialist drug treatment program. Among teachers without prior experience of drug-using students (*n* = 36), the most anticipated action was contacting the parent or guardian (31.0%; *n* = 11). Approximately 80.0% of all teachers (*n* = 76) expressed an interest in receiving further DPE training, with the most frequently cited learning needs as follows: understanding official referral protocols (*n* = 7), detecting drug use among students (*n* = 7), and identifying the most prevalent drug type (*n* = 7).

## 4 Discussion

This study provides the first empirical baseline assessment of DPE-related knowledge, attitudes, and beliefs among in-service male high school PE teachers in Saudi Arabia, based on data collected in 2018, when the Saudi public high school PE workforce was exclusively male. Three findings stand out. First, teachers appear to substantially overestimate their DPE knowledge: despite a mean self-rating of 5.4 out of 7, the objective composite score indicated that approximately half of all pharmacological and legal items were answered incorrectly or left unanswered (M = 1.43/2.00). Item-level data revealed that while legal awareness of DPE was strong, with 95.8% correctly identifying that drug use is a crime, pharmacological knowledge was markedly weaker, with fewer than 40% correctly identifying urine detection windows for cannabis and amphetamine use. These gaps are clinically significant given that Captagon and cannabis are the two most-abused substances by Saudi youth (2, 23).

Prior DPE education was the strongest predictor of both DPE-related knowledge (*t*(88) = −3.45, *p* = 0.001) and attitudes (*t*(87) = 2.6, *p* = 0.011). Despite this, only 30% of teachers had encountered DPE content through college coursework, compared with 58% via the internet and 52% through social media. This discrepancy, namely informal DPE channels outpacing formal DPE training, highlights a structural failure in teacher education to address DPE systematically, and suggests that teachers are actively seeking DPE information that their pre-service programs fail to provide. Years of teaching experience and school sub-district also significantly predicted DPE knowledge: less experienced teachers and those in the Northern district scored higher (*F*(3, 86) = 3.3, *p* = 0.023; *F*(4, 85) = 5.1, *p* = 0.001). This could be accounted for by, first, generational improvements in DPE curriculum content among more recent graduates, and second, geographic inequities in DPE resource provision, both of which warrant targeted policy attention.

Teachers’ attitudes toward DPE were broadly neutral (M = 4.67, SD = 0.95). However, there was strong endorsement of prevention at the policy level (72.6–94.7%), 83.2% supported introducing DPE at the primary level, 84.2% agreed all illegal drugs are harmful to health, and 80.0% believed government investment in drug prevention is worthwhile. That said, a co-existing tension was evident at the practice level, with 30.5% reporting they would avoid teaching a drug-using student and 33.7% saying that doing so would bother them. This divergence between system-level support and student-directed stigma is well documented in drug education research [e.g., Corrigan et al. (24)] and, in the Saudi cultural context where stigma surrounding drug use is particularly pronounced (19), may represent a greater barrier to effective DPE than teachers’ knowledge deficits alone. Notably, more experienced teachers held significantly more positive attitudes toward DPE despite achieving lower DPE-related knowledge scores (*F*(3, 85) = 3.4, *p* = 0.021), suggesting that DPE-related attitudes and knowledge develop along independent trajectories, most likely because direct professional encounters with drug-using students (reported by 63.2% of the sample) foster a pragmatic acceptance of a DPE role among teachers, independent of formal training. This finding implies that pre-service teacher training and CPD must address stigma reduction and attitudinal change through dedicated strategies rather than assuming that knowledge acquisition alone will drive attitudinal improvement in providing DPE.

The most urgent finding concerns the gap between teachers’ stated intentions toward providing DPE and their actual behaviors when encountering a student using drugs. While 81.1% of teachers identified the school counselor as the most appropriate referral target for a drug-using student, in practice, 51.0% of those who had encountered such a student had counseled the student themselves, while 22.0% took no action whatsoever; only one teacher (2.0%) had referred a student to Al-Amal Hospital for specialist treatment. This knowledge-attitude-practice gap (22) indicates that formal referral protocol training is an essential and currently absent component of DPE preparation in Saudi schools. Teachers’ DPE-related curriculum beliefs, by contrast, were strongly positive: all eight National Health Education Standards were endorsed by 72.6–94.7% of respondents, with social and cultural influence content attracting the highest support (94.7%). These results are consistent with the collectivist values that characterize Saudi educational culture (25). Lastly, information-access skills attracted the lowest score (72.6%), likely reflecting the scarcity of validated Arabic-language DPE resources available to teachers and students alike.

The public health implications of the current findings extend far beyond the school gates. Saudi Arabia is currently undergoing a severe and accelerating adolescent drug crisis: 70% of the Kingdom’s drug addicts are under 20 years of age, initiation to drug use commonly occurs in children aged 10–16, and national seizure data place the country among the highest globally for amphetamine-type stimulant use (2, 23). In this context, schools and the PE teachers within them represent one of the few institutional touchpoints with reliable, sustained access to the adolescent population during the developmental window when prevention is most effective (9, 26). PE teachers who lack accurate pharmacological knowledge, hold stigmatizing attitudes toward drug-using students, and bypass specialist referral pathways are not merely failing an important educational objective: they are indirectly contributing to a public health crisis at the population level. The downstream consequences include missed opportunities for early identification and intervention, reinforced stigma that discourages help-seeking among affected adolescents, and a generation of students reaching adulthood without the benefit of evidence-based DPE provision. Addressing the preparedness of PE teachers to provide effective DPE is therefore not a narrow workforce development issue; rather, it is a public health priority with measurable consequences for adolescent morbidity, school safety, and community wellbeing across Saudi Arabia.

It is important to contextualize these findings temporally. The data were collected in 2018, at a time when the Saudi public high school PE workforce was exclusively male. Since then, the Ministry of Education has introduced female PE teachers into public schools (27), and the demographic composition of the PE teaching workforce has changed considerably. Accordingly, the present findings should be interpreted as a historical baseline specific to male PE teachers in Riyadh in 2018, rather than as a description of the current Saudi PE teaching workforce. Comparison of these baseline findings with more recent data, including studies of female PE teachers and teachers from other regions, is an important direction for future research. Notwithstanding these temporal limitations, the structural barriers to DPE identified here (absence of formal curriculum, inadequate pre-service training, and no referral infrastructure) remain relevant to the current Saudi educational context and continue to warrant policy attention.

The results suggest three policy-level actions to improve adolescent DPE provision in Saudi Arabia. First, a skills-based DPE curriculum should be formally embedded in Saudi K-12 schools from the primary level upwards, drawing on evidence-based frameworks such as Life Skills Training (28) and Project Northland (29), contextualized for the Saudi cultural and religious environment. Second, pre-service PE teacher education programs should incorporate a minimum of four to five credit hours of DPE-specific content covering pharmacology, detection, referral, and evidence-based pedagogy. Third, the Ministry of Education should establish a structured annual in-service DPE training cycle in partnership with Al-Amal Hospital and the General Department of Anti-Drugs. This study has three main limitations: first, the cross-sectional design precludes causal inference; second, the sample was restricted to male PE teachers in Riyadh in 2018; third, full psychometric validation of the instrument remains to be conducted. Longitudinal research extending to female teachers and other regions of the Kingdom is a clear and urgent priority for future researchers.

## 5 Conclusion

Based on data collected in 2018, in-service male high school PE teachers in Riyadh were contextually aware of Saudi Arabia’s adolescent drug crisis and broadly supportive of DPE in principle. Yet, they remain structurally underprepared to deliver DPE in practice. Objective knowledge falls far short of perceived competence, professional attitudes retain stigmatizing elements that may impede effective engagement with drug-using students, and responses to such students do not appear to be aligned with evidence-based DPE referral behavior. It is important to note that these failures were not due to the indifference of male Saudi PE teachers; rather, they stem from the absence of systematic DPE preparation at the pre-service and in-service levels. The public health stakes are high: 70% of Saudi drug addicts are aged under 20, and schools operate without any formal DPE curriculum in place, which means the costs of this preparedness gap are borne directly by the adolescent population that PE teachers are uniquely positioned to reach. While the Saudi PE teaching workforce has evolved since 2018, notably with the introduction of female PE teachers, the systemic barriers identified here remain relevant. Coordinated action encompassing curriculum reform, pre-service teacher education, and in-service CPD is both empirically warranted and, given teachers’ expressed readiness to engage in DPE, practically achievable.

## Data Availability

The raw data supporting the conclusions of this article will be made available by the authors, without undue reservation.

## References

[1] Kang J Kim HJ Kim MS Il Shin J Yon DK. Global burden of amphetamine, cannabis, cocaine and opioid use in 204 countries, 1990–2023: a global burden of disease study. Nat Med Nature Publishing Group;. (1990) 32:527–44. doi: 10.1038/s41591-025-04137-0 41545593

[2] UNODC World Drug Report 2025 1st ed Bloomfield: United Nations Research Institute for Social Development (2025) Available online at: https://www.unodc.org/documents/data-and-analysis/WDR_2025/WDR25_B1_Key_findings.pdf (Accessed April 29, 2026).

[3] Altwaijri Y Benjet C Akkad M Bilal L Naseem MT Al-Habeeb A . The epidemiology of substance use disorders in Saudi Arabia: findings from the Saudi national mental health survey. BMC Public Health. (2025) 25:86. doi: 10.1186/s12889-024-21190-5, 39780074 PMC11707954

[4] Ramadan M Alharbi KK. The burden of mental, and substance use disorders in Saudi Arabia: results from the global burden of disease study 2019. BMC Psychiatry. (2019) 25:618. doi: 10.1186/s12888-025-07041-6, 40598028 PMC12211742

[5] Tobaiqy M Al-Asmari AI. Substance misuse disorder in Saudi Arabia: a comprehensive examination of current demographic patterns, trends, and intervention requirements. Saudi Pharm J. (2024) 32:102163. doi: 10.1016/j.jsps.2024.102163, 39262681 PMC11387691

[6] Al-Musa HM Al-Montashri SDS. Substance abuse among male secondary school students in Abha City, Saudi Arabia: prevalence and associated factors. Biomed Res. (2016) 27:1364–73.

[7] Saquib N Rajab AM Saquib J AlMazrou A. Substance use disorders in Saudi Arabia: a scoping review. Subst Abuse Treat Prev Policy. (2020) 15:41. doi: 10.1186/s13011-020-00285-3, 32552804 PMC7301978

[8] Conrod P Stewart SH Seguin J Pihl R Masse B Spinney S . Five-year outcomes of a school-based personality-focused prevention program on adolescent substance use disorder: a cluster randomized trial. Am J Psychiatry. (2025) 182:473–82. doi: 10.1176/appi.ajp.20240042 39810554

[9] Faggiano F Vigna-Taglianti FD Versino E Zambon A Borraccino A Lemma P. School-based prevention for illicit drugs use: a systematic review. Prev Med. (2008) 46:385–96. doi: 10.1016/j.ypmed.2007.11.012, 18258289

[10] Saha P Muhammed JK Chatterjee B Dhawan A. Developmentally tailored school-based interventions for substance use disorders: a narrative review. Indian J Psychiatry. (2026) 68:255–64. doi: 10.4103/indianjpsychiatry_815_25, 41924495 PMC13037823

[11] McKenzie TL Lounsbery MAF. Physical education teacher effectiveness in a public health context. Res Q Exerc Sport. (2013) 84:419–30. doi: 10.1080/02701367.2013.844025, 24592772

[12] Sallis JF Patterson TL Buono MJ Nader PR Patterson TL Buono MJ. Relation of cardiovascular fitness and physical activity to cardiovascular disease risk factors in children and adults. Am J Epidemiol. (1988) 127:933–41. doi: 10.1093/oxfordjournals.aje.a114896 3358413

[13] Ennis CD. Knowledge, transfer, and innovation in physical literacy curricula. J Sport Health Sci. (2015) 4:119–24. doi: 10.1016/j.jshs.2015.03.001, 26558137 PMC4637172

[14] Green K. Physical education, lifelong participation and ‘the couch potato society’1. Phys Educ Sport Pedagogy. (2004) 9:73–86. doi: 10.1080/1740898042000208133

[15] Sallis JF Floyd MF Rodríguez DA Saelens BE. Role of built environments in physical activity, obesity, and cardiovascular disease. Circulation. (2012) 125:729–37. doi: 10.1161/CIRCULATIONAHA.110.969022, 22311885 PMC3315587

[16] AlYahya IA Almohsen HA AlSaleem IA Al-Hamid MM Arafah AM Al Turki YA . Assessment of knowledge, attitude, and practice about first aid among male school teachers and administrators in Riyadh, Saudi Arabia. J Fam Med Prim Care. (2019) 8:684–8. doi: 10.4103/jfmpc.jfmpc_316_18, 30984695 PMC6436304

[17] Nabors LA Little SG Akin-Little A Iobst EA. Teacher knowledge of and confidence in meeting the needs of children with chronic medical conditions: pediatric psychology’s contribution to education. Psychol Sch. (2008) 45:217–26. doi: 10.1002/pits.20292

[18] Rietdijk W. Byrne J. Pickett K. Shepherd J. Roderick P. Grace M. Teachers as health promoters: a longitudinal study of the impact of pre-service teacher training in health education (2015)

[19] AlMarri TSK Oei TPS Al-Adawi S. The development of the short Muslim practice and belief scale. Ment Health Relig Cult. (2009) 12:415–26. doi: 10.1080/13674670802637643

[20] Bryan A. Moran R. Farrell E. O’Brien M. Drug-related knowledge, attitudes and beliefs in Ireland: report of a nation-wide survey Drug Misuse Research Division, Health Research Board (2000). Available online at: https://www.academia.edu/download/38956723/Drug-RelatedKnowledgeAttitudesAndBeliefsInIrelandReportOfANationwideSurvey__.pdf (Accessed 3 May 2026)

[21] SHAPE America-Society of Health and Physical Educators. National Health Education Standards. Champaign: Human Kinetics (2024).

[22] Midford R Cahill H Foxcroft D Lester L Venning L Ramsden R . Drug education in victorian schools (DEVS): the study protocol for a harm reduction focused school drug education trial. BMC Public Health. (2012) 12:112. doi: 10.1186/1471-2458-12-112, 22321131 PMC3305421

[23] Aldlgan AA Hakeem IJ Alandes MN Alfahmi MM. Alcohol and substance abuse in Riyadh, Saudi Arabia: a hospital -based survey. Arab J Forensic Sci Forensic Med. (2019) 1:1421–6. doi: 10.26735/16586794.2019.032

[24] Corrigan PW Kuwabara SA O’Shaughnessy J. The public stigma of mental illness and drug addiction: findings from a stratified random sample. J Soc Work. (2009) 9:139–47. doi: 10.1177/1468017308101818

[25] Botvin GJ Griffin KW. School-based programmes to prevent alcohol, tobacco and other drug use. Int Rev Psychiatry Abingdon, England. (2007) 19:607–15. doi: 10.1080/09540260701797753, 18092239

[26] World Health Organization Global status report on alcohol and health and treatment of substance use disorders (2026) Available online at: https://www.who.int/publications/i/item/9789240096745 (Accessed 14 May 2026)

[27] Saudi Vision 2030 National Transformation Program (2030) (2020) Available online at: https://vision2030.gov.sa/en (Accessed 14 Apr 2019)

[28] Botvin GJ Baker E Dusenbury L Botvin EM Diaz T. Long-term follow-up results of a randomized drug abuse prevention trial in a white middle-class population. JAMA. (1995) 273:1106–12. doi: 10.1001/jama.1995.03520380042033 7707598

[29] Perry CL Williams CL Veblen-Mortenson S Toomey TL Komro KA Anstine PS . Project northland: outcomes of a communitywide alcohol use prevention program during early adolescence. Am J Public Health. (1996) 86:956–65. doi: 10.2105/ajph.86.7.956, 8669519 PMC1380436

